# Integrating pathogen- and host-derived blood biomarkers for enhanced tuberculosis diagnosis: a comprehensive review

**DOI:** 10.3389/fimmu.2024.1438989

**Published:** 2024-08-09

**Authors:** Zhaodong Li, Yunlong Hu, Wenfei Wang, Fa Zou, Jing Yang, Wei Gao, SiWan Feng, Guanghuan Chen, Chenyan Shi, Yi Cai, Guofang Deng, Xinchun Chen

**Affiliations:** ^1^ Guangdong Key Laboratory of Regional Immunity and Diseases, Department of Pathogen Biology, Shenzhen University School of Medicine, Shenzhen, China; ^2^ Guangdong Key Laboratory for Biomedical Measurements and Ultrasound Imaging, National-Regional Key Technology Engineering Laboratory for Medical Ultrasound, School of Biomedical Engineering, Shenzhen University Medical School, Shenzhen, China; ^3^ National Clinical Research Center for Infectious Disease, The Third People's Hospital of Shenzhen, Southern University of Science and Technology, Shenzhen, China; ^4^ Department of Preventive Medicine, School of Public Health, Shenzhen University, Shenzhen, China; ^5^ Guangdong Key Lab for Diagnosis & Treatment of Emerging Infectious Diseases, Shenzhen Third People's Hospital, Shenzhen, China

**Keywords:** tuberculosis, mycobacterium tuberculosis, biomarker, blood test, molecular diagnosis

## Abstract

This review explores the evolving landscape of blood biomarkers in the diagnosis of tuberculosis (TB), focusing on biomarkers derived both from the pathogen and the host. These biomarkers provide critical insights that can improve diagnostic accuracy and timeliness, essential for effective TB management. The document highlights recent advancements in molecular techniques that have enhanced the detection and characterization of specific biomarkers. It also discusses the integration of these biomarkers into clinical practice, emphasizing their potential to revolutionize TB diagnostics by enabling more precise detection and monitoring of the disease progression. Challenges such as variability in biomarker expression and the need for standardized validation processes are addressed to ensure reliability across different populations and settings. The review calls for further research to refine these biomarkers and fully harness their potential in the fight against TB, suggesting a multidisciplinary approach to overcome existing barriers and optimize diagnostic strategies. This comprehensive analysis underscores the significance of blood biomarkers as invaluable tools in the global effort to control and eliminate TB.

## Introduction

Tuberculosis (TB), caused by Mycobacterium tuberculosis (Mtb), remains a formidable public health challenge globally. In 2022, it was responsible for 1.3 million deaths worldwide, underscoring its persistent threat despite medical and public health advancements (1). The situation is further aggravated by the emergence of multidrug-resistant TB (MDR-TB), which poses severe hurdles to eradication efforts, particularly in regions with dense populations and limited healthcare infrastructure (2–5). To address these challenges, the World Health Organization (WHO) launched its End Tuberculosis 2035 strategy, aiming to reduce TB’s prevalence and burden (6). A critical aspect of this strategy, outlined in the WHO’s 2014 consensus report, is the development of a triage test with high negative predictive value (NPV) to confidently rule out disease in negative cases and reduce unnecessary testing (7). Furthermore, systematic screening is required, with the test having at least 90% sensitivity and 70% specificity, usable by first-contact clinicians to identify patients needing further testing (7).

**Figure 1:**
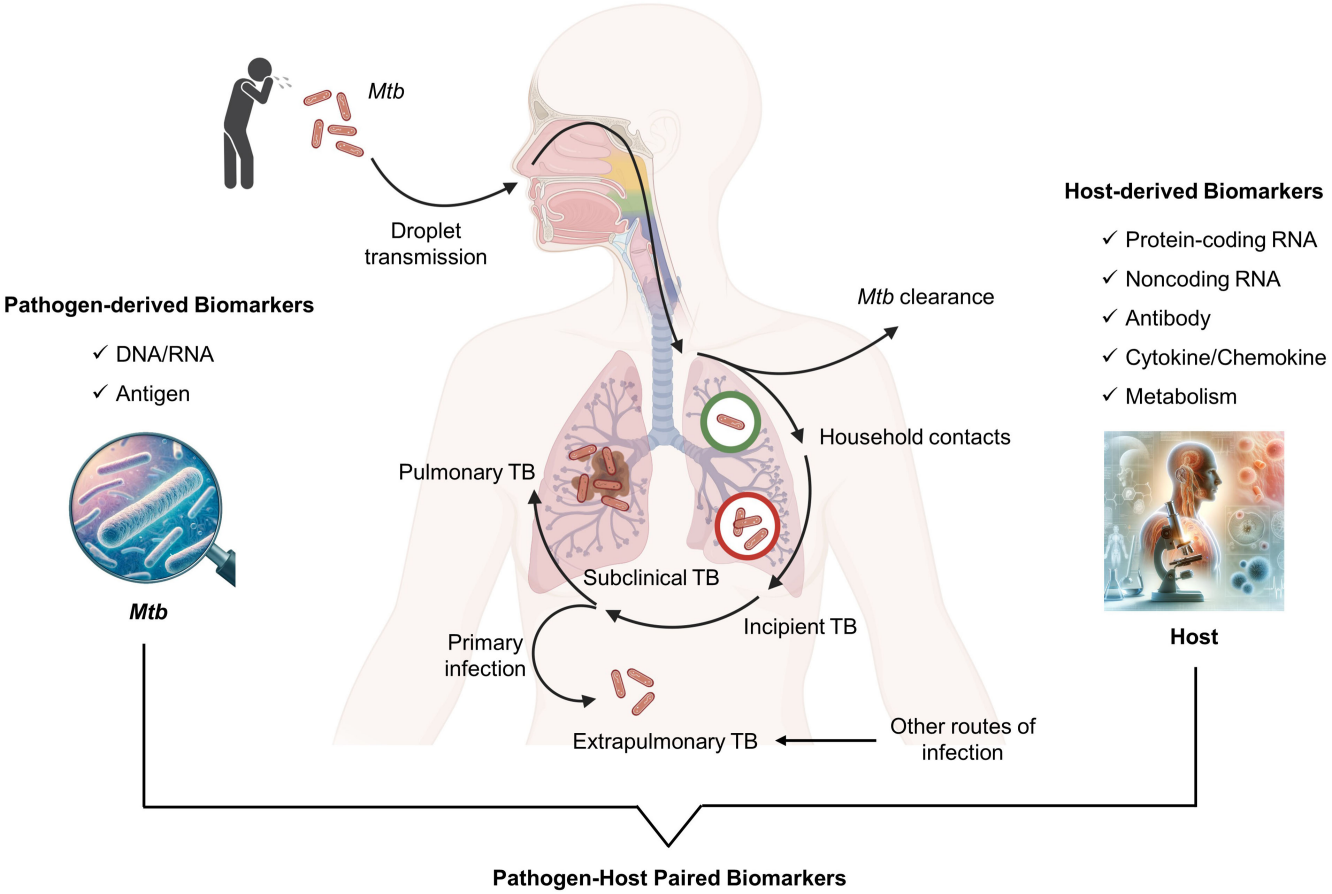
Category of pathogen and host derived biomarkers in blood for TB diagnosis.

The dynamics of TB infection result from a balance between the host’s immune system and Mtb. In addition to the established states of disease and infection, there are two distinct intermediate clinical phases: incipient TB and subclinical TB (**Figure 1**). Incipient TB involves bacterial replication within affected tissues and a partial breakdown of the immune response, without any clinical, radiological, or microbiological evidence of disease. It’s believed that without therapeutic intervention, this incipient stage of the disease could evolve into active TB disease (8, 9). Subclinical TB, while symptom-free, shows increased Mtb replication and tissue damage detectable through microbiological assays and radiological imaging (8, 10). However, some individuals can control the infection at the incipient and subclinical phases by inducing robust immune responses, preventing progression to active TB (11). This highlights the critical role of the host’s immune system in managing TB infection and the potential for early intervention strategies to prevent disease progression to active TB. Accurate diagnosis of TB infection is vital for preventing disease progression and providing preventive therapy, a key strategy in global TB control (12).

TB diagnostics has evolved significantly over the years, featuring a variety of methods each developed to address specific needs and challenges associated with the disease’s detection. For example, solid medium Mtb culture, which is highly specific but with variable sensitivity, is less practical for quick decision-making as it takes up to 8 weeks to confirm the result. Liquid medium Mtb culture using Mycobacteria Growth Indicator Tube system, although reducing processing time to 2–3 weeks and improving sensitivity to 80–90%, demands high setup costs and advanced lab facilities (13–16). Sputum-smear microscopy is quick and cost-effective but less sensitive and specific, particularly in low-bacterial-load cases (17–19). Tuberculin skin test (TST) is inexpensive and simple but less specific in BCG-vaccinated individuals and requires a follow-up visit (20–22). Interferon gamma release assays (IGRA) provide a more specific alternative to TST with results in 24 hours, though costs are higher, limiting use in constrained budgets (23). GeneXpert MTB/RIF, detecting Mtb DNA and rifampicin resistance in two hours, suits point-of-care use with its minimal lab needs, but its high cost may restrict widespread adoption (24). Additionally, Line Probe Assays and Whole Genome Sequencing (WGS) can offer precise genetic data for diagnosing and guiding treatment but are expensive and require sophisticated labs, limiting their use to well-resourced healthcare settings or complex cases (25).

In summary, limitations of these methods primarily involve around their accessibility, cost, and operational requirements, which can be prohibitive in regions most affected by TB. Furthermore, the need for specialized training for accurate operation and interpretation of results adds another layer of complexity, restricting their utility in lower-resource settings. Therefore, it is in great need to develop more accessible, cost-effective, and rapid diagnostics, such as novel blood biomarkers, which could potentially transform TB detection globally. Here, we review the current findings and diagnostic potential of blood biomarkers, and explore their implications for improving TB detection globally.

## Overview of blood biomarkers of tuberculosis

In the current landscape of TB diagnosis, blood biomarkers are categorized into those derived from the pathogen and the host (**Figure 1**). Pathogen-derived biomarkers include specific mycobacterial DNA or RNA sequences and antigens detectable in blood, such as microbial cell-free DNA (cfDNA), IS6110, IS1081, lipoarabinomannan (LAM), early secreted antigenic target 6 (ESAT-6), and CFP-10. These are primarily used for diagnosing TB disease. On the other hand, host-derived markers typically involve components of the immune response, such as antibodies (IgG and IgA against specific Mtb antigens), cytokines (IFN-gamma, VEGF, TNF-alpha), metabolites, and transcriptional profiles indicative of Mtb exposure or infection. For instance, recent advances have identified host RNA signatures that can differentiate between TB and control individuals with excellent diagnostic efficacy, boasting AUC values over 0.900 (26, 27). These findings highlight their potential as diagnostic biomarkers for detecting TB disease. Thus, the blood biomarkers, both pathogen and host-derived, could be useful for a more tailored approach to manage TB.

## Pathogen-derived biomarkers

### Nucleic acid biomarkers

Application of Polymerase Chain Reaction (PCR) and real-time PCR in nucleic acid amplification tests (NAATs) are typical methods for directly detecting TB by targeting specific DNA or RNA sequences unique to Mtb (28, 29). However, the sensitivity of PCR for directly detecting Mtb DNA in blood samples is notably low, ranging from 0.304 to 0.490 (30, 31). Developments such as the GeneXpert MTB/RIF and GeneXpert MTB/RIF Ultra have been introduced to diagnose TB rapidly with high specificity but have shown limited sensitivity, particularly in immunocompromised patients (32–34). Additionally, these tests are typically used on non-blood samples from individuals suspected of having TB, indicating that they are not directly comparable in the context of blood-based TB biomarkers. To address these gaps, there is a critical need to innovate and enhance PCR technologies to improve the accuracy of TB diagnosis using blood samples. Emerging techniques such as digital PCR (dPCR) and droplet digital PCR (ddPCR) show promise for achieving this goal (27, 35–37).

The dPCR testing method, which combines PCR with microfluidic technology, accurately measures low levels of nucleic acid targets. A previous study has exhibited the ability of dPCR quantifying targets IS6110 (dPCR-IS6110) and IS1081 (dPCR-IS1081) in discriminating TB individuals from healthy volunteers with AUCs of 0.790 and 0.720, respectively, and they had a promising specificity (0.934) but diminished sensitivity (<0.450) (36), which was further proved by another publication (38). Additionally, dPCR was used to determine Mtb complex DNA in PBMCs, revealing that 156 out of 197 (79%) PBMCs were detectable (39). Droplet Digital PCR (ddPCR), developed based on dPCR, subdivides the sample into thousands or even millions of tiny, independent droplets, each containing a small fraction of the total sample (35). Characterized by high sensitivity, precision, and robustness against inhibitors, this method allows for absolute quantification without the need for standard curves, making it particularly effective for analyzing complex clinical samples (40). By quantifying IS6100 copy numbers using ddPCR, Yang et al. reported that Mtb DNA can be detected Mtb in all examined full blood samples from both pulmonary TB cases (28/28) and extrapulmonary TB patients (28/28) (40). Besides IS6110, CFP-10 also serves as an amplification target for ddPCR, showing promising performance in TB diagnosis with 100% sensitivity and specificity (41). Besides TB diagnosis, ddPCR has also been applied to vaccine efficacy evaluation (41).

Microbial cell-free DNA (cfDNA) circulating in the bloodstream has garnered substantial attention due to its potential as a promising biomarker for detecting infections (42, 43). The microbial CRISPR system is an essential tool for detecting cfDNA. It first targets specific DNA or RNA sequences by designing particular guide RNAs and then cuts these targets with CRISPR effector enzymes such as Cas9, Cas12a, and Cas13, followed by the amplification and identification of the cut fragments. Therefore, combination of CRISPR system and isothermal amplification has established a CRISPR-based diagnostic (CRISPR-Dx) technology, providing rapid DNA or RNA detection. For example, Gootenberg JS, et al. have developed a Cas13a-based molecular detection platform named Specific High-Sensitivity Enzymatic Reporter UnLOCKing (SHERLOCK), which can detect single specific RNA and DNA targets to identify particular strains of Zika and Dengue virus, distinguish pathogenic bacteria, and detect human genotype DNA, but it was not initially applied to detecting Mtb (44). Subsequently, Gootenberg JS, et al. optimized and upgraded SHERLOCK (termed SHERLOCK-V2), adding Cas12a and the CRISPR type III effector nuclease Csm6 to the original single-channel detection to expand it to four channels, enhancing the signal sensitivity by 3.5 times (45). SHERLOCK-V2 holds a promising diagnostic platform with optimal accuracy for TB detection, although it has not been available for TB.

In 2023, Thakku SG, et al. evaluated the effectiveness of SHERLOCK in detecting Mtb, showing a detection limit of 0.5–5 fg of fragmented DNA and a compromised positive detection rate of 55% (6/11) (43). To improve the sensitivity of SHERLOCK, Thakku SG, et al. applied the SHERLOCK to conduct pooled amplification and subsequently simultaneously identified multiple target molecules tiled across the pathogen genome based on CRISPR-Cas13 detection, which was a novel method called Wholegenome Assay using Tiled Surveillance Of Nucleic acids (WATSON). WATSON improves the detection limit by 10- to 100-fold (0.05–0.5 fg) and achieves a 91% positive identification rate (10/11) in the TB cohort (43). Additionally, Huang Z, et al. developed an ultra-sensitive fluorescence CRISPR-Cas12a detection technique (CRISPR-TB) for identifying Mtb-cfDNA in blood, facilitating TB diagnosis (42). In an HIV-negative cohort, CRISPR-TB demonstrated a sensitivity of 0.960 and a specificity of 0.940 for detecting TB in adults, and a sensitivity of 0.830 and specificity of 0.950 in children. In HIV-positive cohorts, it achieved an overall TB detection rate of 85%, with a 100% detection rate in pediatric cases (42). Beyond TB, cfDNA/CRISPR-Dx holds potential for broader applications in detecting various infectious diseases and monitoring conditions.

### Antigen biomarkers

Mtb antigens can trigger detectable immune responses in human hosts. The most commonly utilized antigen for TB diagnostics is lipoarabinomannan (LAM) found in urine, especially among HIV-positive patients, and in pleural fluid (46, 47). Additionally, Mtb LAM in blood also serves as a biomarker for diagnosing TB. For instance, Brock M and colleagues have developed a method using the Single Molecule Array (Simoa) to detect serum LAM, achieving a sensitivity of 37% with 100% specificity in HIV-positive TB patients. The sensitivity can be 60% in HIV-positive/smear-positive TB patients (48). This method offers perfect specificity but poor sensitivity, insufficient to meet WHO standards, highlighting the need for innovative diagnostic techniques that leverage the unique properties of LAM to improve its sensitivity in blood sample. To this end, Zhang W, et al. developed a nanoparticle-enhanced immunoassay read by dark-field microscopy that detects LAM and its carrier protein on the surface of circulating extracellular vesicles, through which, 74% of HIV-positive children, 73% in cases missed by microbial tests, and 80% in undiagnosed TB cases were positive for LAM test (49).

Another significant antigen-based method is the use of Mtb culture filtrate proteins, such as the early secreted antigenic target 6 (ESAT-6), a major virulence factor of Mycobacterium pathogenic species, secreted in the blood and sputum of infected individuals. ESAT-6 enabled a rapid, specific, and accurate detection at all Mtb infection stages (50). In earlier years, Poulakis N, et al. applied flow cytometry to assess Mtb ESAT-6 levels in the blood of TB patients, accurately diagnosing 90% of TB cases, with sputum culture-positive patients consistently showing ESAT-6 expression (50). More recently, the growing interest in electrochemical sensors for diagnosing Mtb has been noteworthy. For instance, an anti-ESAT-6 monoclonal antibody as a bio-receptor is first covalently immobilized on the surface of a gold-plated screen-printed electrode (SPE) in a label-free electrochemical immunosensor, which has a detection limit of 7ng/ml (51). However, this sensor has not been tested for stability and reusability. Moreover, the cost of SPE sensors for TB diagnosis is prohibitive, especially in developing countries where Mtb is prevalent. Therefore, Omar RA, et al. found a cost-effective electrode substitute (Ni-rGO-PANI) and conducted qualitative and quantitative measurement of ESAT-6 using cyclic voltammetry. This substitute, made from Ni, metal-reduced graphene oxide (rGO), and polyaniline (PANI), not only reduces diagnostic costs but also lowers the detection limit for EAST-6 to 1ng/ml (52). Recently, a new electrochemical aptasensor with MXene/C60NPs/Au@Pt for signal generation and amplification was developed to detect and quantify EAST-6 in blood, with detection limits of 2.88 fg/ml (DPV measurement) and 13.50 fg/ml (IT measurement), respectively (53). It shows optimal potential in distinguishing TB patients from healthy donors and other lung disease patients, with 97.5% sensitivity and 96.7% specificity, suggesting a promising significant diagnostic application in clinic (53).

Additionally, immune-PCR (I-PCR), which is equipped with the characteristics of antigen–antibody interaction in ELISA and the capability of exponential amplification in PCR (54). It has been demonstrated that the sensitivity of I-PCR is much higher than that of ELISA (55). With CFP-10 as the target protein, I-PCR exhibited a sensitivity range of 0.762–0.837 in detecting TB patients, with a specificity of 0.935–0.938 (56). Singh N, et al. had previously stated that in addition to CFP-10, Mtb ESAT-6, CFP-10, MPT64, Ag85B, PstS1, and LAM can also serve as target proteins for I-PCR (57), indicating I-PCR’s potential as a promising tool for Mtb infection. Subsequently, real-time I-PCR has an advantage over conventional I-PCR in minimizing carry-over contamination, and thus it can monitor the dynamic alterations of Mtb secreted proteins in clinical samples, highlighting its potential for monitoring TB progression and treatment responses (58).

**Figure 2:**
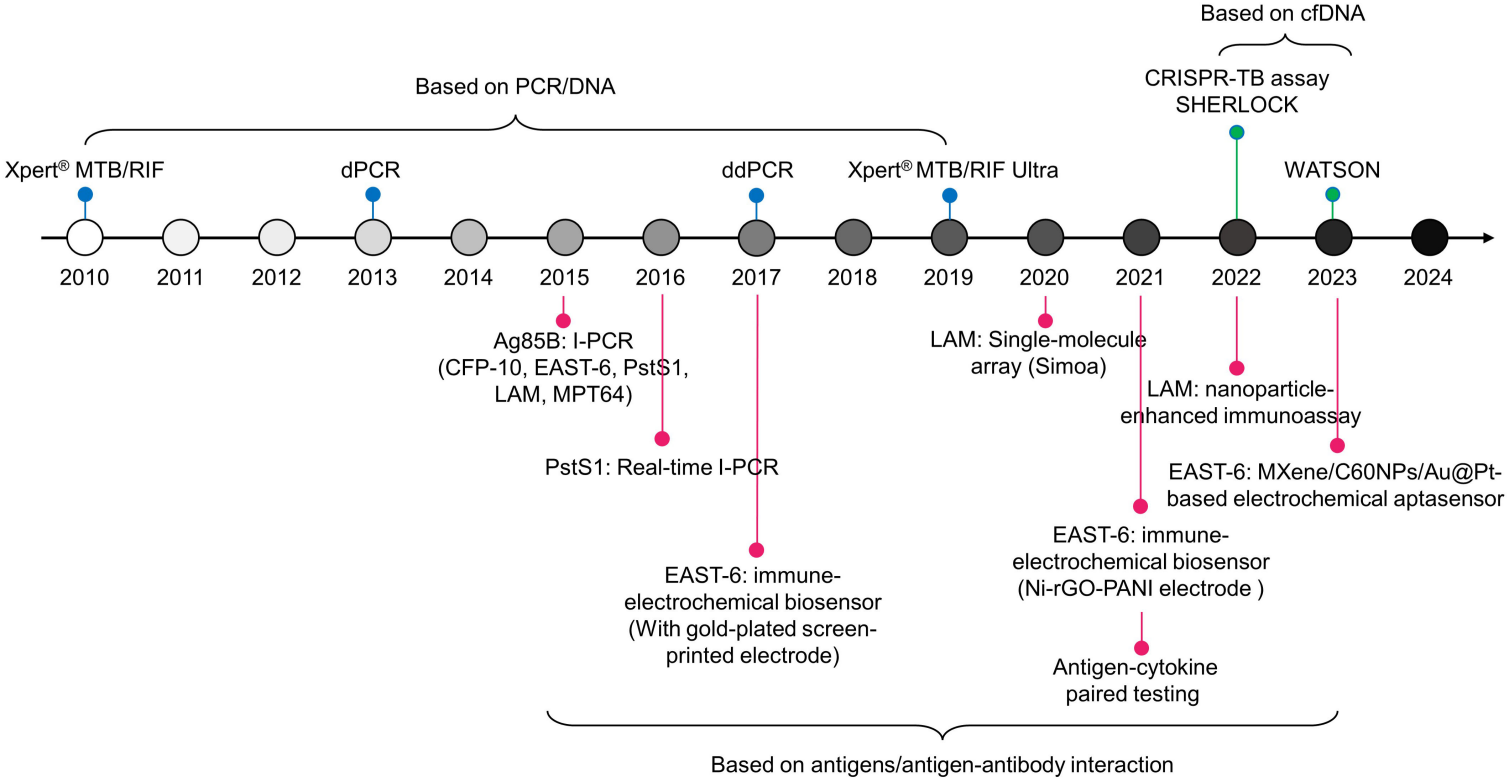
Timeline of TB diagnostic technologies for pathogen biomarkers since 2010.

Recent research suggests that incorporating host cytokines into Mtb antigens will enhance the performance of TB detection (59). Meier NR, et al. identified ten antigen-cytokine pairs among 154 combinations, where the biomarker consisting of these ten pairs exhibited an AUC value of 0.920 in discriminating TB from controls. The most discriminative classifiers included Rv2346/47c-IP-10, Rv3614/15c-IP-10, Rv2031c-GM-CSF, and ESAT-6/CFP-10-TNF-alpha, which were the top four combinations (60). Additionally, Rv2431c-IP-10, Rv2031c-TNF-alpha, Rv2346/47c-TNF-alpha, and Rv2431c-TNF-alpha showed significant differences between TB individuals and control volunteers, with corresponding AUCs of 0.929, 0.953, 0.938, and 0.921, respectively (61). The goal of these combined biomarkers is to enhance the diagnostic performance of TB, especially in cases with atypical clinical presentations or complex infection scenarios, such as in children and HIV-positive populations (60, 61). In practice, development and validation of these combined biomarkers require extensive clinical trials and scientific verification to ensure their accuracy and practicality. To date, the new technologies discussed in this paper for developing markers for Mtb have been summarized in **Figure 2**.

## Host-derived biomarkers

### Messenger RNA biomarkers

One prominent example involves the use of gene signatures, which are combinations of multiple genes whose collective expression levels provide a robust diagnostic indicator. A notable case is a study by Kaforou M, et al., which developed a 27-transcript signature to discriminate TB from LTBI and a 44-transcript signature to distinguish TB from other diseases, with an excellent sensitivity and specificity regardless of HIV status (62). However, large number of transcripts signatures makes them unfeasible as biomarkers for clinical TB diagnosis, especially in point-of-care and resource-limited settings. Sweeney et al. addressed this issue by further refining the diagnostic gene set based on a multicohort study (26). They developed a three-gene signature (Risk3), comprising GBP5, DUSP3, and KLF2, to effectively separate TB individuals from healthy controls (AUC, 0.900), LTBI subjects (AUC, 0.880), and ODs patients (AUC, 0.840). Subsequently, Wu X, et al. quantified the mRNA expression levels of Risk3 in the Shanghai Pulmonary Hospital Cohort to evaluate the signature’s performance in TB detection, which showed a classification ability in distinguishing TB from LTBI, ODs, and healthy volunteers with AUCs of 0.739 (sensitivity 0.597, specificity 0.781), 0.825 (sensitivity 0.821, specificity 0.656), and 0.892 (sensitivity 0.761, specificity 0.880), respectively (63). To some extent, these results suggest that Risk3 has potential as a rapid, blood-based screening and triage test for TB. Another transcript signature, comprising GBP1, IFITM3, P2RY14, and ID3, was developed based on forest models and confidence interval decision trees, discriminating between TB and healthy controls with an AUC of 0.910 in the HIV-negative cohort (64). However, HIV co-infection could decrease the performance of the signature with an AUC of 0.840 (64), which contrasts with the viewpoints of Kaforou, M, et al. and Sweeney et al. (26, 62). The contrasting viewpoints may be due to cohort heterogeneity, model-building methods, and sample processing techniques.

**Figure 3:**
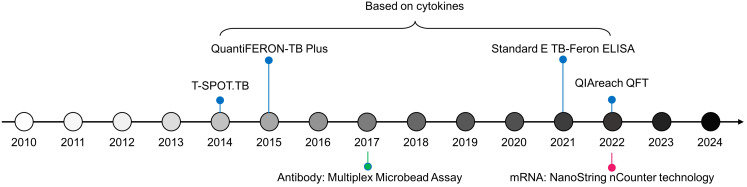
Timeline of TB diagnostic technologies for host biomarkers since 2010.

Based on the signatures from Karorou M, et al., Gliddon HD, et al. reduced the number of transcripts using feature selection algorithms and further quantified the expression levels of these characteristics using dPCR (27). Therefore, a 4-transcript signature (GBP6, TMCC1, PRDM1, and ARG1), derived from the 44-transcript signature (62), distinguishes TB from other diseases with an AUC of 0.938; a 3-gene transcript (FCGR1A, ZNF296, and C1QB), derived from the 27-transcript signature (62), separates TB from LTBI with an AUC of 0.973 (27). Additionally, using NanoString nCounter technology, Kaipilyawar V, et al. developed a NanoString 6-gene set (NANO6), comprising CCR6, BLK, DARS2, EXOC2, ATP1A1, ANKRD22, which accurately distinguishes TB from LTBI with an AUC of 0.987, sensitivity of 1.000, and specificity of 0.932 (65), which is a powerful method providing robust and precise quantification of mRNA based on color-coded barcode probes to directly measure gene expression levels without the need for amplification. Besides, this tool offers optimal accuracy and ability to simultaneously analyze multiple targets, holding promising prospects in clinical applications. The novel technologies for developing markers for host discussed in this paper are summarized in **Figure 3**.

In recent years, Ho J, et al. established two early diagnostic models for tuberculosis (TB) (11). Initially, they identified patients who were Xpert MTB positive and MTB sputum culture positive and/or had a chest x-ray (CXR) but lacked symptoms typical of active pulmonary TB, as early-stage TB patients. They then developed a 7-gene signature and an 8-gene signature, the former to distinguish TB from uninfected individuals (AUC=0.856), and the latter to differentiate TB from latent TB (AUC=0.892) (11). Establishing an early diagnosis model for TB is crucial because it enables timely initiation of treatment, reducing transmission and improving patient outcomes. Additionally, Sivakumaran D, et al. classified the TB disease process into four stages: household contacts, incipient TB, subclinical TB, and active TB (8). They developed a TB diagnostic biomarker based on 11 immune-related genes (11-gene signature) to distinguish between these stages (active TB vs. subclinical TB, AUC=1.000; active TB vs. household contacts, AUC=0.860; subclinical TB vs. household contacts, AUC=0.990), and it can differentiate between progressive TB and incipient TB patients (AUC=0.820) (8). This differentiation aids in tailoring interventions, monitoring disease progression, and implementing preventive strategies effectively, thereby reducing the overall burden of TB and preventing outbreaks within communities.

### Non-coding RNA biomarkers

It’s reported that 80% of DNA is transcribed into RNA, and only 1.5% of this RNA is translated into protein, suggesting cellular RNA consists of two distinct forms: non-coding RNA (ncRNA) and protein-coding RNA (66). The potential of host-derived non-coding RNAs, specifically microRNAs (miRNAs), as biomarkers for TB diagnosis has drawn considerable attention. One key miRNA identified is miR-155, which was recognized as early as 2012 for its potential as a TB diagnostic marker (67). Both Mycobacterium bovis BCG infection and ROS are known to upregulate miR-155 expression (68), and it is involved in immune responses mediated by macrophages and T-cells (69). Daniel EA, et al. reviewed 21 studies to perform a meta-analysis evaluating miRNAs’ potential as TB biomarkers, showing the performance of miR-155 in TB diagnosis with a sensitivity of 0.898 and specificity of 0.809 (70). Diagnostic performance of miR-31, an inflammation-related factor, surpasses that of miR-155, exhibiting a sensitivity of 0.960 and a specificity of 0.890 (70, 71). Additionally, Shepelkova GS, et al. utilized miR-155 to characterize the pathological states of TB (72), demonstrating that miR-155 distinguishes TB patients without tissue lesions from healthy controls with a sensitivity of 0.730 and specificity of 0.999; and distinguishes TB individuals with tissue lesions from those without, with a sensitivity of 0.909 and specificity of 0.929, offering new insights for TB diagnosis and treatment. However, the small number of cases used in this study means that the accuracy and stability of the biomarkers could not be thoroughly evaluated.

Recently, Gunasekaran H, et al. conducted a meta-analysis derived from seven studies, revealing that both miR-197 and miR-144 exhibited higher sensitivity and specificity in differentiating TB from healthy controls, with sensitivities of 0.935 and 0.953, and specificities of 1.000 and 0.991, respectively (73), highlighting two potentially valuable miRNA candidates. Additionally, miR-29a-3p and miR-361–5p showed higher sensitivity in distinguishing TB from LTBI, with sensitivities of 0.905 and 0.887, but with compromised specificities of only 0.622 and 0.439, respectively (73). A 3-miRNA signature, comprising miR-197–3p, miR-let-7e-5p, and miR-223–3p, was developed to diagnose DR-TB with a sensitivity of 1.000 and specificity of 0.750 (73). The 3 candidate miRNAs identified by Gunasekaran H, et al. were inconsistent with another 3-miRNA signature developed by Liang Q, et al. (74). Liang Q, et al. combined miR-506–3p with miR-543 and miR-195–5p and developed a new 3-miRNA signature to distinguish spinal TB (STB) from healthy controls with a better AUC of 0.902 (74). Particularly, the miRNA signature had significantly higher risk scores in patients with STB compared to those with pulmonary TB and other spinal diseases, demonstrating the miRNA signature’s potential to differentiate STB from other conditions (74).

Circular RNA (circRNA), known for its stability and tissue specificity, is involved in many pathological and physiological processes in individuals with tuberculosis (TB), offering a promising avenue for biomarkers in TB diagnosis and assessing the risk of TB progression. Both hsa_circ_0001953 and hsa_circ_0009024 are significantly increased in individuals with TB compared to healthy controls and are positively associated with radiological scores, suggesting that both circRNAs may be involved in TB severity (75). Hsa_circ_0001953 has good performance in TB diagnosis with an AUC of 0.856, sensitivity of 0.740, and specificity of 0.900, followed by hsa_circ_0009024 with an AUC of 0.808, sensitivity of 0.700, and specificity of 0.800 (75). Moreover, the combination of hsa_circ_0001953 and hsa_circ_0009024, a 2-circRNA signature, achieves an AUC of 0.915. Meanwhile, this 2-circRNA signature shows promising potential to distinguish TB from other diseases (75). The circRNAs biomarkers for TB diagnosis by Huang Z, et al. are completely different from the characteristic circRNAs identified by Qian Z, et al. (76). Qian Z, et al. developed a 7-circRNA signature to detect TB with an improved AUC of 0.946. Meanwhile, Fu Y, et al. consider circRNA_103017 (AUC=0.870) to be a key candidate circRNA for TB diagnosis, followed by circRNA_059914 (AUC=0.821) and circRNA_101128 (AUC=0.817) (77). Additionally, the corresponding AUCs for circRNA_051239, circRNA_029965, and circRNA_404022 were 0.974, 0.944, and 0.968, respectively, in distinguishing TB from healthy controls (78). The reasons for these inconsistencies might due to the following reasons. First, the differences sample used, which mainly include whole blood, peripheral blood, serum, PBMCs, and plasma; Second, variations in detection methods, primarily comprising whole-genome sequencing, RNA sequencing, microarray, qRT-PCR, and dPCR. Third, the differences in the selection methods and criteria for candidate core genes, mainly reflected in the thresholds set for logFC and p-value (or adjusted p-value).

Dysregulation of long non-coding RNAs (lncRNAs) has been demonstrated to be closely involved in various pathological and physiological processes by regulating innate and adaptive responses. In recent years, lncRNAs in host blood have gained prominence as potential biomarkers for TB diagnosis. For example, a diagnostic lncRNA signature consisting of four lncRNAs (NR_038221, NR_003142, ENST0000057036, and ENST00000422183) was reported to discriminate TB from healthy controls with an AUC of 0.845, a sensitivity of 79.2%, and specificity of 75% (79). LOC152742 was also regarded as a biomarker for TB diagnosis with a better AUC value of 0.923 in discriminating active TB from healthy individuals, and an AUC of 0.852 among active TB and obsolete TB patients (80), which was the only publication for a biomarker used to distinguish between active and obsolete TB patients. Differing from the above in sample source, Huang S, et al. directly used neutrophils to screen for lncRNAs as TB diagnostic biomarkers, namely lncRNA ZNF100–6:2, with a good AUC value of 0.980 (81). However, this study did not specify which kind of individuals were used to distinguish TB for achieving the reported AUC (81). In addition to comparisons between TB and healthy controls, lncRNAs as diagnostic biomarkers for cured TB have been reported, such as a combined lncRNA signature comprising uc.48+ and NR_105053 with an AUC of 0.945 (sensitivity 0.900, specificity 0.864) (82). Due to limitations in sample size, different populations, and lack of external validation, it is challenging to ensure the consistency and validity of lncRNA biomarkers in TB diagnostic outcomes. Perhaps the following method could address this issue.

lncRNAs can also be combined with electronic health record (EHR) metrics to form lncRNA-EHR biomarkers (83–85). The diagnostic AUC of lncRNA-EHR biomarkers for TB ranges from 0.850 to 0.900. EHR data can significantly enhance the diagnostic efficiency and clinical applicability of disease diagnostic models (86). These EHR metrics primarily include age, hemoglobin, cough, weight loss, low-grade fever, calcification detected by computed tomography [CT calcification], and the interferon gamma release assay for tuberculosis [TB-IGRA]. Besides lncRNAs, circRNAs, miRNAs, transcripts, and proteins can all be integrated with EHR for diagnosing TB and other diseases, which is increasingly becoming a new focus of research in disease diagnostics.

### Antibody-based methods for TB diagnosis

An ideal TB diagnostic biomarker should be simple, able to be measured on-site, capable of rapid testing, and discern across different stages of TB (87). A growing body of research suggests that antibodies fulfill these criteria due to their low cost, ease of use, and versatile functionality throughout the various stages of TB infection (87, 88). Currently, three main types of antibody-based tests are used in TB diagnosis: monoclonal antibodies, polyclonal antibodies, and combined antibodies targeting single and/or multiple Mtb antigens (87). Antibody-based tests targeting a single Mtb antigen typically show variable and sometimes suboptimal sensitivity (ranging from 0.290 to 0.940), but they maintain high specificity (ranging from 0.844 to 1.000) in distinguishing TB from non-TB controls (89, 90). Examples of such Mtb antigens include mammalian cell entry protein 1A (Mce1A), PstS1, proline-proline-glutamic acid protein 17 (PPE17), A60, Ag85, HspX, CFP-10, CFP-21, ESAT-6, and MPT-64 (89–92).

To address the issue of low sensitivity associated with antibody-based tests, innovative testing tools like the Multiplex Microbead Assay and Protein Chip Array have been developed for TB identification. These tools have demonstrated optimal sensitivity (0.900–0.940), though their specificity remains suboptimal (0.770–0.890) (93). Moreover, combinations of Mtb antibodies have been shown to achieve optimal sensitivity and specificity in TB diagnosis. Awoniyi DO and colleagues utilized Anti-Tpx+L16 IgG, anti-Tpx IgG, and anti-MPT64 IgA to develop a multi-antibody-based test that distinguished TB individuals from control volunteers with a sensitivity of 0.952 and a specificity of 0.976 (94). Another study reported a 7-antibody-associated test including IgA antibodies against Rv3019c, PstS, Apa, NarL, MPT64, Tpx, and the 19 kDa antigen, along with an IgM antibody against LAM. This test differentiated TB from other respiratory diseases with an AUC of 0.800, a sensitivity of 0.654, and specificity of 0.769 (95). Furthermore, the application of antibodies in the clinical diagnosis of TB has been well summarized (40, 87, 96).

### Cytokine biomarkers

The importance of IFN-gamma as the first biomarker used for TB diagnosis cannot be overstated. Its role in detecting Mtb infection has paved the way for the development of various diagnostic tests, such as the QuantiFERON-TB Gold In-Tube test (QFT-GIT) (97, 98) and T-SPOT.TB assay (99), which utilize IFN-gamma release assays (IGRAs) to measure serum levels of Mtb-specific interferon-gamma (IFN-gamma) release (100). Meanwhile, IFN-gamma quantification remains the gold standard for detecting Mtb infection (101).

In 2015, QIAGEN upgraded the QFT-GIT to QuantiFERON-TB Plus (QFT-plus). Based on a meta-analysis involving 12 publications, Sotgiu G, et al. demonstrated that the corresponding values of AUC, sensitivity, and specificity were 0.990, 0.940, and 0.960, respectively, for QFT-plus in discriminating between TB and healthy controls (102). Compared to QFT-GIT (sensitivity 0.910), the sensitivity of QFT-plus was higher in detecting TB (102), which was also demonstrated by Chien JY, et al. (QFT-plus vs. QFT-GIT, 1.000 vs. 0.894) (103). Additionally, among the cured TB cases, the sensitivity of QFT-plus in TB diagnosis was also higher than QFT-GIT (0.820 vs. 0.730) (104). The promising sensitivity of QFT-plus mainly resulted from the TB2 responses. QFT-GIT only had a TB1 tube containing ESAT-6 and CFP-10 antigens to quantify the IFN-gamma from CD4+ T-cells (105). However, QFT-plus included another antigen tube, TB2, and was designed to measure IFN-gamma production by both CD4+ and CD8+ T-cells, improving sensitivity in diagnosing TB particularly among individuals with compromised immunity (106).

The T-SPOT.TB assay, which utilizes the enzyme-linked immunospot (ELISPOT) technique, quantifies IFN-γ-producing T-cells in response to Mtb-specific antigens (ESAT-6 and CFP-10) to detect Mtb infection (107). This assay demonstrated sensitivity values of 0.910 for detecting TB peritonitis and 0.780 for meningitis, with specificities of 0.780 and 0.680, respectively (108, 109). Additionally, it showed excellent performance in diagnosing TB among rheumatic patients, with a sensitivity of 0.930 and specificity of 0.940 (110), highlighting its potential in clinical practice. However, similar to QFT-Plus, the T-SPOT.TB assay requires advanced laboratory equipment and is more costly compared to the tuberculin skin test (TST).

On the other hand, IGRAs have various limitations, such as higher false-positive and false-negative rates (111), incomplete separation between TB and LTBI (112), and a lack of appropriate cut-off criteria for positive cases (113). It is urgent to develop novel testing tools based on new Mtb-specific antigens and new immunodiagnostic biomarkers to overcome the problems associated with IGRAs, such as Standard E TB-Feron ELISA and QIAreach QFT. Both of the IFN-gamma-testing tools have demonstrated excellent diagnostic efficacy and cost-effectiveness in detecting TB disease (114).

In addition to IFN-gamma, TNF-alpha, IL-2, and IL-17A have been identified as biomarkers for TB detection (115). Kumar NP and colleagues found that a combination of these three cytokines (TNF-alpha, IL-2, and IL-17A) formed a signature that was more effective in distinguishing TB from non-TB individuals than any individual marker or any combination of two markers (115). Furthermore, a current publication developed a support vector machine (SVM) model by combining IFN-gamma with TNF-alpha, IL-6, and IL-10, measuring serum levels of these four cytokines, which held a potential as biomarker for diagnosing TB with an AUC of 0.850, a sensitivity of 0.850 and specificity of 0.780 (101). Notably, this model outperformed the IGRA among HIV-infected volunteers (101). As an Mtb-specific biomarker, VEGF showed significantly different expression levels between TB and LTBI patients, underscoring its diagnostic capabilities (116–118). Additionally, chemokines such as CXCL9/MIG and CXCL10/IP-10 were reported to be significantly upregulated in TB patients compared to uninfected volunteers (119, 120). Meanwhile, a diagnostic model based on MIG and IP-10 demonstrated promising performance in differentiating TB from healthy individuals, achieving a sensitivity of 1.000 and a specificity of 0.950 (120).

### Metabolic biomarkers

Upon Mtb infection, the metabolite dynamics resulting from the interaction between Mtb and the host play crucial roles in the innate immune response and in regulating the host’s defense system (121), indicating their potential as biomarkers for TB diagnosis. A recent publication identified 8 candidate metabolites for detecting TB based on a random forest algorithm, of which the most powerful classifying efficacy was LysoPE (18:1(11Z)/0:0) with an AUC of 0.995, a sensitivity of 1.000, and specificity of 0.963, followed by (2R)-O-Phospho-3-sulfolactate (AUC, 0.990; sensitivity, 0.933; specificity, 0.963) and 8-Demethyl-8-formylriboflavin 5′-phosphate (AUC, 0.986; sensitivity, 1.000; specificity, 0.889) (122). However, the study did not assess the overall classifying performance of the 8 candidates. Additionally, aside from active TB, Li YX, et al. have also used LC-MS/MS to identify Inosine, 16, 16-dimethyl-6-keto Prostaglandin E1, Theophylline, and Cotinine as potential serum indicators for Mtb latent infection and the metabolites were involved in amino acid metabolism, the endocrine system, the immune system, and lipid metabolism (123). Meanwhile, Wang X, et al. also identified seven metabolites as biomarkers to distinguish LTBI from healthy controls (124). Unlike Li YX, et al. who screened for metabolic biomarkers, Wang X, et al. not only assessed the diagnostic efficacy of each metabolite but also combined the 7 markers into a signature with an AUC of 0.979 (124).

## Future perspectives and research directions

One of the main challenges of using host biomarkers for disease diagnostics is their lack of specificity. Host biomarkers often represent general immune responses rather than responses specific to a particular pathogen, leading to false positives and difficulties in distinguishing between different infections or inflammatory conditions. For instance, biomarkers such as C-reactive protein (CRP) and various cytokines (e.g., IL-6, TNF-α) are elevated in many infectious and inflammatory diseases, not just tuberculosis (TB) (125, 126). This lack of specificity can result in misdiagnosis or delayed diagnosis, as elevated levels of these biomarkers do not unequivocally indicate TB infection. Conditions such as autoimmune diseases, other bacterial, viral, and fungal infections, as well as chronic inflammatory conditions, can also cause similar changes in host biomarker levels, necessitating the use of a combination of biomarkers and additional diagnostic tests to improve accuracy (100, 127, 128). Recent research has focused on identifying more specific host biomarkers for TB, such as IP-10 and certain TB-specific T-cell responses (129, 130); however, these markers still require further validation and testing in diverse populations to ensure their reliability and specificity. In summary, while host biomarkers hold great promise for non-invasive and rapid diagnostics, their lack of specificity remains a significant hurdle. Ongoing research and the development of more specific biomarkers, along with multi-modal diagnostic approaches, are essential to overcome these challenges and improve the accuracy of TB diagnostics.

**Figure 4:**
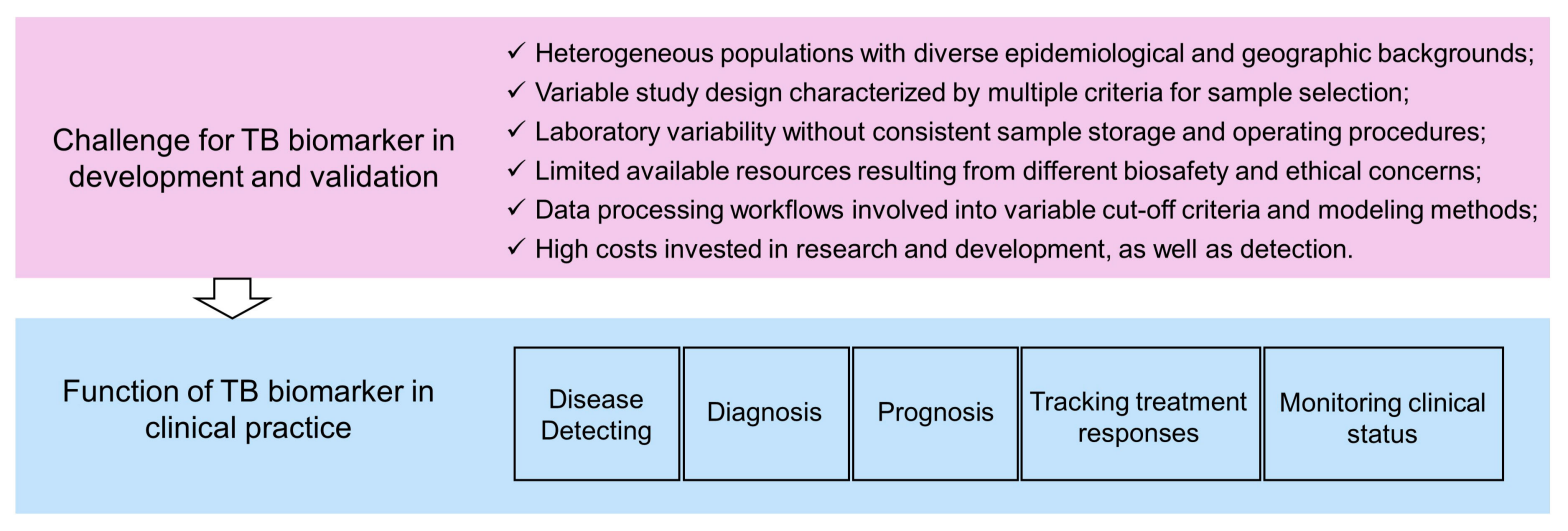
Challenge and function for a promising TB diagnostic biomarker.

An ideal biomarker for TB detection should meet at least the following two requirements. First, it has excellent sensitivity and specificity, using either binary classification or quantified data. Second, it should be suitable for routine clinical tests with the properties of a short detection cycle, ease of operation, easily accessible samples, and low testing costs (131). Host blood diagnostic testing is undoubtedly a very convenient method, among which genetic testing is most common. Current molecular biomarkers applied in blood tests for TB patients face a general challenge: they have ideal sensitivity and specificity in internal validation cohorts (training cohort and testing cohort), but show diminished diagnostic efficacy in external validation cohorts (independent dataset). For this reason, Nogueira BMF, et al. have suggested that heterogeneous populations, variable study design, laboratory variability, limited available resource, and the different data processing workflow contribute to inconsistent results (**Figure 4**) (133. Particularly, they have proposed solutions for the corresponding limitations (132).

The journey of a biomarker from discovery to clinical application is complex process, involving multiple phases such as discovery, validation, and eventual integration into clinical practice (131). Advancements in technologies like single-cell next-generation sequencing (133), liquid biopsies for circulating targets (134), and other high-throughput methods (135–137) have significantly accelerated biomarker discovery by generating vast amounts of data quickly and cost-effectively. Challenges remain in the effective analysis and use of this data, particularly in harnessing electronic health records for biomarker-driven healthcare (131, 138). Additionally, when evaluating multiple biomarkers, it’s crucial to control for multiple comparisons, using metrics such as the false discovery rate, to enhance the reliability of the findings. This careful evaluation, which includes assessing associations with disease status and demographic or clinical features, informs the design of validation studies and ultimately aids in establishing robust biomarkers across different stages of disease management (131).

In clinical oncology and medicine, definitions for levels of evidence help assess biomarkers’ clinical utility, but challenges like bias and missing data often complicate validation efforts (139). Bias, particularly, arises from various sources during a study such as patient selection, specimen handling, and analysis, which can skew results away from the truth. Techniques like randomization and blinding are critical for mitigating bias; they ensure unbiased distribution and evaluation of specimens and data across study groups (140, 141). Additionally, it’s crucial to have a robust plan for addressing missing data in studies, which includes identifying why data are missing and implementing strategies to minimize the impact of such missingness on the study results (131). These measures are essential to preserve the integrity and reliability of biomarker research. Additionally, to enhance diagnostic accuracy, leveraging a panel of multiple biomarkers typically outperforms a single biomarker. Using variable selection methods like shrinkage helps minimize overfitting and improves validation likelihood (142). For complex models involving interactions or advanced techniques like machine learning, generating pilot data for simulations is crucial to guide sample size estimations and develop an effective analytical approach (143, 144).

## Conclusion

In conclusion, the investigation into blood biomarkers for tuberculosis (TB) diagnosis reveals a promising avenue towards enhancing early detection and accurate monitoring of the disease. The review underscores the significance of both pathogen-derived and host-derived biomarkers in providing critical insights into TB’s pathophysiology and the host’s immune response. These biomarkers are critical for detecting TB at various stages of infection and in different patient populations, including those co-infected with HIV and those suffering from ETB. Pathogen-derived biomarkers offer direct indicators of TB presence, while host-derived markers are essential for understanding the immune dynamics and could guide treatment decisions. Additionally, the work advocates for a more integrated approach in clinical practice, combining these biomarkers with traditional diagnostic methods to develop a more comprehensive and accurate TB diagnostic strategy, offering insights into the host-pathogen interactions and disease pathology. Despite the potential, the review acknowledges challenges such as the need for comprehensive validation and standardization across diverse clinical environments to ensure the biomarkers’ efficacy and reliability. The integration of advanced analytical tools and multidisciplinary approaches is recommended to overcome these barriers, facilitating the transition from traditional methods to more precise and predictive diagnostics. This could significantly impact global TB management, reducing the disease burden by enabling timely and targeted therapeutic interventions.
